# MARINE AND COASTAL SCIENCE: Faster Test for Detecting Contamination of Recreational Waters

**DOI:** 10.1289/ehp.118-a201a

**Published:** 2010-05

**Authors:** Carol Potera

**Affiliations:** **Carol Potera**, based in Montana, has written for *EHP* since 1996. She also writes for *Microbe*, *Genetic Engineering News*, and the *American Journal of Nursing*

Swimmers and surfers face the risk of contracting gastrointestinal illnesses from exposure to water contaminated with human sewage. The current method for monitoring fecal indicator bacteria (FIB) in recreational waters requires collecting water samples, then culturing and counting microbes in the laboratory, a process that takes 24 hours. This delay may expose swimmers to tainted water or, conversely, unnecessarily close beaches that are no longer contaminated. Now engineers at the University of California, Los Angeles (UCLA) have designed a better rapid-detection method that directly analyzes FIB onsite in recreational waters in less than 1 hour.

Called the covalently linked immunomagnetic separation/adenosine triphosphate (Cov-IMS/ATP) technique, the portable process uses magnetic beads linked covalently to antibodies that bind FIB. The bead-captured FIB are ruptured and treated with an enzyme (luciferase) that catalyzes a light-emitting reaction powered by ATP. A luminometer measures the amount of light emitted, which correlates with bacterial concentrations.

UCLA’s Jennifer Jay, an associate professor of civil and environmental engineering, graduate student Christine Lee, and coworkers collected ocean samples from a California beach and from freshwater streams that flow into the beach area. They checked for two common FIB—*Escherichia coli* and *Enterococcus*. The Cov-IMS/ATP method correctly identified 87% of *E. coli* and 94% of *Enterococcus* in the samples, producing results similar to standard culture-based methods performed for comparison. Moreover, the new method detected FIB at limits below what the U.S. Environmental Protection Agency deems healthy for recreational waters. The findings were published online 24 December 2009 ahead of print in the *Journal of Applied Microbiology*.

Other efforts to develop rapid tests for recreational water quality are based on the quantitative polymerase chain reaction (for example, see *EHP* 114:24–28 [2006]). A field test based on Cov-IMS/ATP would be easier to use, according to Mark Gold, president of Heal the Bay, an environmental group in Santa Monica, California, that monitors aquatic habitats. That’s because quantitative polymerase chain reaction takes about 3 hours and requires cumbersome equipment, plus samples must be transported to a laboratory.

Now the UCLA team is adapting the method to identify *Bacteroidales* species, microbes that can be definitively linked specifically to human fecal pollution. “*E. coli* and *Enterococcus* are not ideal fecal indicators because they do not tell you the source of the fecal pollution, and they grow naturally in the environment,” says Jay. In contrast, bacteria in the *Bacteroidales* family grow only in the intestines of warm-blooded animals, with different species targeting different animals. These bacteria also do not replicate well in the environment. So the detection of *Bacteroidales* signals recent fecal pollution. “Even more important,” says Jay, “you can tell whether *Bacteroidales* comes from humans or an animal to target cleanup efforts.” The team’s measurements of FIB in freshwater streams to test whether the method could track beach pollution to a particular storm drain will be submitted for publication separately.

The Cov-IMS/ATP method could potentially become a tool for beach managers to analyze water samples in the morning and post public health warnings within a few hours. Gold says, “This would protect public health better than the current system, where beaches are closed based on yesterday’s results.”

